# The relationship between stroke mortality and red blood cell parameters

**Published:** 2014-10-06

**Authors:** Hamidreza Hatamian, Alia Saberi, Matin Pourghasem

**Affiliations:** 1 Department of Neurology, School of Medicine, Guilan University of Medical Sciences, Rasht, Iran; 2 Department of Pediatrics, School of Medicine, Guilan University of Medical Sciences, Rasht, Iran

**Keywords:** Stroke, Anemia, Mortality

## Abstract

**Background: **Several factors influence on the outcome of ischemic stroke. The aim of this study was determination the relationship between stroke mortality and red blood cell parameters.

**Methods:** This cross-sectional study was conducted from 2011 July to June 2012. For all patients with ischemic stroke in middle cerebral artery (MCA) territory, the cell blood count test was performed. We recorded their mortality on the 1^st^ week and the 1^st^ month after ischemic stroke. Data analysis was performed using t-test, χ^2^, Mann–Whitney U-test, logistic regression and receiver operating characteristic curve in SPSS for Windows 19.0.

**Results: **A total of 98 subjects (45.9% men and 54.1% women) with the mean age of 71.0 ± 13.9 years were assessed, while 67.3% of them were anemic. The prevalence of 1^st^ week mortality among anemic and non-anemic patients was 40.9% and 34.4% (P = 0.534). The prevalence of mortality after 1^st^ week till 1^st^ month was 19.6% and 21.0% respectively (P = 0.636). In univariant analysis, only 1^st^ month mortality had a significant relationship with red blood cell (RBC) count (P = 0.022). However, the result of logistic regression model showed that RBC (P = 0.012) and mean corpuscular volume (MCV) (P = 0.021) remained as predictors of the 1^st^ week and the 1^st^ month mortality (P = 0.011 and P = 0.090 respectively). The best cutoff point of RBC for the prediction of the 1^st^ week mortality with 44.7% specificity and 69.5% sensitivity was estimated 4.07 million/μl and for the 1^st^ month mortality with 46.6% specificity and 72.2% sensitivity was estimated 4.16 million/μl.

**Conclusion: **The RBC count and MCV are independent predictors of ischemic stroke short-term mortality.

## Introduction

Stroke is one of the disabling disorders and the third cause of death in developed countries.^1^ Its ischemic type contributes to 85% of all.^2^ Several factors including anemia is known as a risk factor for stroke.^2^^-^^4^

During an ischemic stroke, a cascade of cellular and molecular events starts.^2^ Numerous factors including anemia may impact on its development and cause different prognosis of ischemic stroke in the same arterial territory.^5^ In addition; it is suspected that anemia influences on the patient’s resistance and compensation for surveillance. In addition, the disturbance in oxygenation of the other organ can lead to multiple organ failure and death.

In this project, we aimed to determine if anemia is associated with stroke mortality? In addition, we were interested to determine the cutoff point of red blood cell (RBC) indices for prediction of stroke mortality.

## Materials and Methods

This cross-sectional study carried out in an educational hospital of the Guilan University of Medical Sciences (GUMS), Iran, in 2011-2012 with achievement the confirmation of the ethic committee of GUMS.

For omitting the confounding effect of the arterial territory, only middle cerebral artery (MCA) territory infarction was included. The patients who involved by ischemic stroke in this territory participated in the study after fulfilling an informed consent by their legal responsible. The involvement of MCA was ascertained by the brain computed tomography scan. The patients whose medical files were incomplete or MCA involvement were not established were excluded. The treatment was identical for all of them, and no additive expenditure was imposed on patients. The patients’ information and RBC count and its indices including mean corpuscular volume (MCV), mean corpuscular hemoglobin (MCH), mean corpuscular hemoglobin concentration (MCHC), hemoglobin (Hb) and hematocrit (HCT) and their 1^st^ week and 1^st^ month mortality by any cause were recorded. Anemia was defined as two standard deviation lower than means of Hb and HCT values for each sex groups. Hence that HCT < 36.0% and Hb < 12.0 g/dl among women and HCT < 41.0% and Hb < 13.5 g/dl among men were considered as anemia.^5^

The sample volume was determined as 101 patients according to 7 as reference number, with confidence of 99% and the test power of 95%.

Kolmogorov–Smirnov (KS) test, parametric t-test or non-parametric Mann–Whitney U and χ^2^ tests, receiver operating characteristic (ROC) curve and multiple logistic regression models in SPSS for Windows 19.0 (SPSS Inc., Chicago, IL, USA) software were used for analysis of data.

## Results

From 101 patients who participated; 98 patients with a mean age of 71.0 ± 13.9 (25-103) years old (45.9% men and 54.1% women) completed this study.

About 67.3% of them were anemic with a mean age of 70.9 ± 15.2 years old with no significant difference with non-anemic ones (17.3 ± 11.0 years) (P = 0.887).

The 1^st^ week mortality rates among anemic and non-anemic patients were 40.9% and 34.3% (P = 0.534). The 1^st^ month mortality rates were 60.6% and 56.0% (P = 0.636), that 19.6% of anemic and 21.0% of non-anemic patients were passed away between 1^st^ week and 1^st^ month after stroke.

In univariant analysis, the association between 1^st^ week or 1^st^ month mortality rate and all studied parameters was not significant (P > 0.050) except between 1^st^ month mortality and RBC count (P = 0.022) (Table 1).

The maximum predictive areas for the 1^st^ week [area under curve (AUC) = 0.604 0.057] and the 1^st^ month (AUC = 0.611 0.059) mortality were close to being significant only for RBC count.

In multivariant analysis, only RBC count [P = 0.210, odds ratio (OR) = 0.385 (95% confidence interval (CI): 0.184-0.808)] and MCV [P = 0.012, OR = 0.841 (95% CI: 0.893-0.991)] remained as the 1^st^ week mortality predictors and RBC count [P = 0.011, OR = 0.422 (95% CI: 0.217-0.822)] and age [P = 0.016, OR = 1.042 (95% CI: 1.077-1.008)] as the 1^st^ month mortality predictors.

**Table 1:** Comparison the means of hemoglobin, hematocrit, red blood cell, mean corpuscular volume, mean corpuscular hemoglobin, and mean corpuscular hemoglobin concentration between dead and alive patients with ischemic stroke patients

**Red blood cell parameters**	**First week**		**First month**	
	**Mean ** **± ** **SD**	**P**	**Mean ** **± ** **SD**	**P**
Hb				
Alive	12.2 ± 2.1	0.200	11.8 ± 1.9	0.255
Dead	11.7 ± 1.7		12.2 ± 2.1	
HCT				
Alive	37.7 ± 5.8	0.156	36.4 ± 4.6	0.107
Dead	36.2 ± 4.1		38.2 ± 5.9	
RBC				
Alive	4.5 ± 0.9	0.223	4.3 ± 0.7	0.022
Dead	4.2 ± 0.6		4.6 ± 0.9	
MCV				
Alive	83.9 ± 10.1	0.748	80.8 ± 15.7	0.407
Dead	78.2 ± 17.2		83.1 ± 9.5	
MCH				
Alive	27.7 ± 6.6	0.619	27.3 ± 4.1	0.750
Dead	27.1 ± 4.0		27.6 ± 7.5	
MCHC				
Alive	32.1 ± 1.8	0.936	32.1 ± 1.9	0.955
Dead	32.1 ± 1.7		32.1 ± 1.7	

Hb: Hemoglobin; HCT: Hematocrit; RBC: Red blood cell; MCV: Mean corpuscular volume; MCH: Mean corpuscular hemoglobin; MCHC: Mean corpuscular hemoglobin concentration; SD: Standard deviation

The cutoff point for prediction of the 1^st^ week mortality with 44.7% specificity and 69.5% sensitivity was estimated as 4.07 million/μl [likelihood ratio (LR+) = 1.26, LR− = 0.68] and for the 1^st^ month mortality with 46.6% specificity and 72.2% sensitivity was estimated as 4.16 million/μl (LR+ = 1.37, LR− = 0.59). 

## Discussion

In the present study, the prevalence of anemia based on WHO criteria was 67.3% in the present study that is near the maximum reported prevalence among geriatrics in a systematic review of the literature by Beghe et al. (from 2.9% to 61.0% in elderly men and from 3.3% to 41.0% in elderly women).^6^

The stroke mortality in the 1^st^ week was 40.9% and 34.3% among anemic and non-anemic patients respectively. In spite of 6% difference, the result was not statistically significant. After 1 month, 60.6% and 56.0% of anemic and non-anemic patients were dead that 19.6% and 21.0% of them were dead between the 1^st^ week and the 1^st^ month that this difference was not significant also. Whereas, in Tanne et al. study, that was performed in 2001-2002; after 1 month, the mortality rate was 23.9% and 13.2% among anemic and non-anemic patients [OR = 1.90 (95% CI: 1.5-3.43)].^7^ In another study by Nybo et al., anemia was introduced as a predictive factor of stroke mortality that both of them are in disagreement with our results.^8^ About the comparison of mortality rate in our study that was higher than the others and also than the population-based study in Iran by Borhani-Haghighi et al.^9^ and WHO MONICA Project;^10^ the difference may be due to not only the difference of emergency and hospital care, but also the contribution of all types of arterial territory involvement in their study, not similar to ours which only involved by ischemic stroke in the MCA territory with high mortality rate.

Although in our study by univariant analysis, the 1^st^ week mortality rate has no significant association with all of the RBC indices but the first month mortality was related to RBC count. It can be explained that the 1^st^ week mortality is related to the nature of ischemic stroke and some events such as herniation, whereas the 1^st^ month mortality is related to another factors such as patients’ compensation that may be related to anemia and RBC indices.

According to logistic regression analysis, only RBC count and MCV remained as predictors of the 1^st^ week mortality and RBC and age only account for 1^st^ month mortality. With increasing one unit of RBC count, the 1^st^ week and the 1^st^ month mortalities decrease 3.0 and 2.5 folds respectively. In addition, with increasing one unit of MCV measure, the 1^st^ week mortality decreases 6.0%. In ROC curve, the maximum predictive area for mortality was estimated only for RBC count. The best cutoff point of RBC count for the 1^st^ week mortality was 4.06 million/μl. It seems that its specificity is low (44.7%) and sensitivity is moderate (69.5%). And for the first month mortality was estimated 4.16 million/μl. It seems that its specificity is also low (46.6%), however its sensitivity is high (72.5%). The LR+ and LR− of cut-off point were 2.26 and 0.68 in the 1^st^ week and 1.35 and 0.59 in the 1^st^ month. Although all of them were close to 1, but the measures of 1 month became farther than 1.

## Conclusion

It seems that we can use RBC count and its cutoff point and eventually MCV measurement as the predictors of short-term mortality of ischemic stroke.

## Notes

**How to cite this article: **Hatamian H, Saberi A, Pourghasem M. The relationship between stroke mortality and red blood cell parameters. Iran J Neurol 2014; 13(4): 237-40.
